# Free Skin Grafting to Reconstruct Donor Sites after Radial Forearm Flap Harvesting: A Prospective Study with Platelet-Rich Fibrin (PRF)

**DOI:** 10.3390/jcm11123506

**Published:** 2022-06-17

**Authors:** Anton Straub, Roman Brands, Anna Borgmann, Andreas Vollmer, Julian Hohm, Christian Linz, Urs Müller-Richter, Alexander C. Kübler, Stefan Hartmann

**Affiliations:** 1 Department of Oral and Maxillofacial Plastic Surgery, University Hospital Würzburg, Pleicherwall 2, 97070 Würzburg, Germany; brands_r@ukw.de (R.B.); borgmann_a@ukw.de (A.B.); vollmer_a@ukw.de (A.V.); hohm_j@ukw.de (J.H.); linz_c@ukw.de (C.L.); mueller_u2@ukw.de (U.M.-R.); kuebler_a@ukw.de (A.C.K.); hartmann_s2@ukw.de (S.H.); 2 Comprehensive Cancer Center Mainfranken, University Hospital Würzburg, Josef-Schneider-Str. 6, 97080 Würzburg, Germany; 3 Bavarian Cancer Research Center (BZKF), 91054 Erlangen, Germany

**Keywords:** platelet-rich fibrin, free skin grafts, radial forearm flap, donor-site morbidity

## Abstract

Reconstruction of the donor site after radial forearm flap harvesting is a common procedure in maxillofacial plastic surgery. It is normally carried out with split-thickness or full-thickness free skin grafts. Unfortunately, free skin graft transplantation faces wound healing impairments such as necrosis, (partial) graft loss, or tendon exposure. Several studies have investigated methods to reduce these impairments and demonstrated improvements if the wound bed is optimised, for example, through negative-pressure wound therapy or vacuum-assisted closure. However, these methods are device-dependent, expansive, and time-consuming. Therefore, the application of platelet-rich fibrin (PRF) to the wound bed could be a simple, cost-effective, and device-independent method to optimise wound-bed conditions instead. In this study, PRF membranes were applied between the wound bed and skin graft. Results of this study indicate improvements in the PRF versus non-PRF group (93.44% versus 86.96% graft survival, *p* = 0.0292). PRF applied to the wound bed increases graft survival and reduces impairments. A possible explanation for this is the release of growth factors, which stimulate angiogenesis and fibroblast migration. Furthermore, the solid PRF membranes act as a mechanical barrier (“lubrication” layer) to protect the skin graft from tendon motion. The results of this study support the application of PRF in donor-site reconstruction with free skin grafts.

## 1. Introduction

With its versatile character and success rates of over 95%, the radial forearm flap has become a standard procedure in the reconstruction of tissue defects in the head and neck region [1]. It is a fasciocutaneous microvascular flap, which is particularly suitable for intraoral use. Closure of the donor site is consequently a procedure that is performed just as frequently, normally using free skin grafting [2]. Unfortunately, reconstruction of the donor site faces wound healing impairment in up to 30% of patients [3,4,5]. Vascular in-growth is crucial to graft survival and healing [6]. However, this is often compromised by the movement of the tendons and poor wound-bed conditions, which can lead to tendon exposure and (partial) graft loss [3,4,5]. Furthermore, pain, infection, and graft adherence to the tendon are associated with healing problems [2]. Thus, conditioning the wound bed prior to free skin graft transplantation is essential to the closure of these donor sites. Some authors have described techniques such as the use of full-thickness instead of split-thickness skin grafts, negative-pressure wound therapy, or vacuum-assisted closure to improve healing [3,5,7]. Full-thickness skin grafts are mechanically more resistant than split-thickness skin grafts and thus cover the tendons and prevent their exposure more effectively, albeit suffering from an elevated risk of insufficient blood supply [4,5,8]. Any method that can increase the mechanical resistance without compromising the blood supply would clearly be a promising approach to improve graft in-growth [4,5,8]. One such method, the placement of platelet-rich fibrin (PRF) membranes between the wound bed and the skin graft, may fulfil both these criteria.

PRF is an autologous blood concentrate, which is prepared by centrifuging the patient’s blood. It supports wound healing by releasing growth factors such as platelet-derived growth factor, vascular endothelial growth factor, and various interleukins, which have a positive effect on angiogenesis and fibroblast migration [9]. Furthermore, inflammatory cells (for example, leukocytes, monocytes, and macrophages) that are likewise present in PRF are also known to accelerate wound healing [10,11,12,13]. Depending on the preparation protocol, PRF is either liquid and suitable for injection, or solid and firm in the form of membranes [9]. The release of growth factors and inflammatory cells is similar in both preparations [14,15]. These features led to the widespread use of PRF in the field of oral and maxillofacial surgery, particularly at sites of damage caused by osteonecrosis or in complex bone augmentations [16]. Nevertheless, prospective trials studying different possible indications for PRF application are lacking.

Several studies, for example, Clark in 2019, investigated the benefits of optimising the wound bed on surgical outcomes in the reconstruction of radial forearm donor sites [3]. This preconditioning improved graft healing significantly and reduced healing complications. Overall, Clark et al. demonstrated that a well-prepared wound bed increases graft survival. Unfortunately, most of these methods are device-dependent, expansive, and time-consuming.

We hypothesise that the benefits of PRF, when applied between the wound bed and skin graft, are similar to negative-pressure wound therapy or vacuum-assisted closure. The release of various growth factors, as described above, may improve vascular in-growth and transplant survival, as well as reduce the frequency of dehiscence and other complications such as infection or necrosis. Furthermore, the solid membranes may increase the mechanical resistance of the skin graft to tendon motion. This additional layer, acting as a lubricant between the tendons and skin graft, could therefore reduce mechanical stress and tendon exposure [8]. This would improve surgical outcomes, independent of the method of skin grafting implemented (full-thickness versus split-thickness). To the best of our knowledge, this is the first study to date to investigate the effects of PRF on the healing and survival of free skin grafts in the reconstruction of the donor site after harvesting a radial forearm flap.

## 2. Materials and Methods

**Figure 1:**
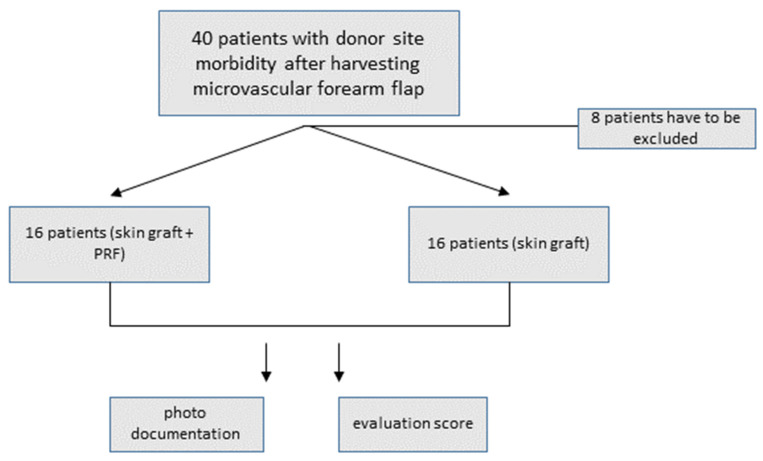
Flowchart depicting patient selection and study design.

A prospective clinical evaluation of 32 patients who underwent free skin graft reconstruction of the donor site following microvascular forearm flap harvesting was performed in which the participants were randomised into two groups. In group 1, the wound bed and tendons of the forearm were covered with PRF prior to skin transplantation. In group 2, the skin graft was placed directly onto the wound bed (non-PRF). Patients were enrolled between October 2020 and September 2021 in the Clinic of Maxillofacial and Plastic Surgery in Würzburg (see Figure 1). The institutional review board of the University of Würzburg approved the study protocol (approval reference number 143/20-me).

The inclusion criteria were donor-site morbidity following microvascular forearm flap surgery, reconstruction of this lesion with a free skin graft, an age of 18 or older, and the provision of informed consent. The skin graft was obtained from either the thigh, the abdomen, or the arm. The surgeon was responsible for the selection of the donor region for the skin graft. Pressure bandages and sutures were removed after ten to twelve days (T1). At this time, the site was documented photographically using a scale bar and an evaluation score was determined. Patients were excluded when inclusion criteria were not matched or when failures in the study protocol or lost follow-up occurred (e.g., no photo documentation or evaluation score after ten to fourteen days).

### 2.1. Free Skin Graft Harvest

**Figure 2:**
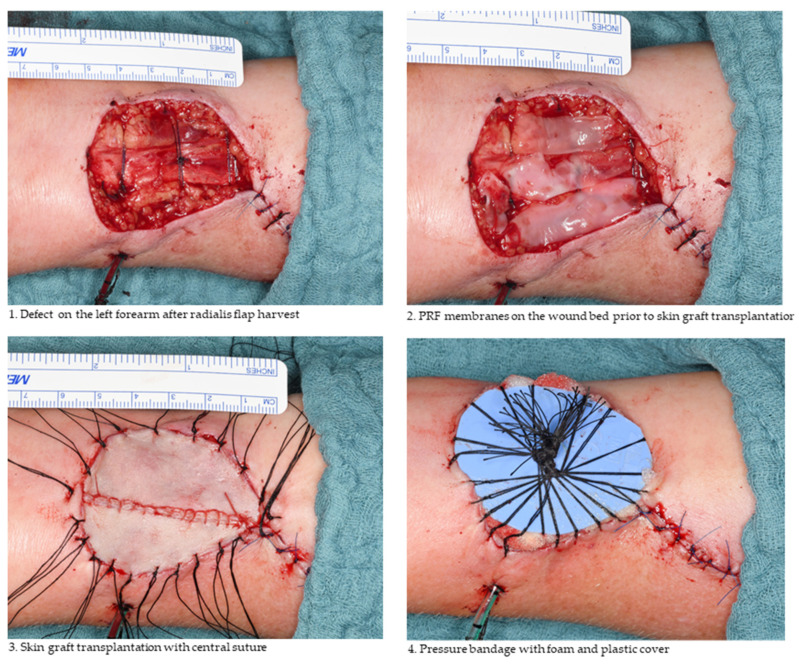
(**1**) Illustrates the defect on the forearm after radial forearm flap harvest. (**2**) Wound bed and tendons were covered with PRF membranes. (**3**) Full-thickness free skin graft from the ipsilateral arm was placed on the PRF membranes. (**4**) Pressure bandage was applied and followed by sterile dressing.

In both groups, defects on the forearm were reconstructed with free skin grafts either immediately after forearm flap removal (primarily) or later on in a second operation. Full-thickness skin grafts were typically harvested from the abdomen or ipsilateral arm, whereas split-thickness skin grafts normally originated from the thigh. The surgical procedure was similar in both groups and only differed in the use of PRF to cover the wound bed and tendons in group 1 (see Figure 2). Experienced maxillofacial surgeons performed the forearm flap harvest and tried to preserve the peritenon in all patients.

Split-thickness skin grafts were harvested with a dermatome with a layer thickness of 0.4 mm. After transplantation, a pressure bandage was applied with the use of foam and a plastic cover (see Figure 2). To reduce the risk of dehiscence, in some cases, two to three minor perforations were performed on the graft depending on the operator’s choice. We did not use an immobilisation splint in any case.

### 2.2. Platelet-Rich Fibrin Preparation

PRF was prepared using a Mectron platelet-rich fibrin centrifuge (A-PRF Duo centrifuge, Mectron, Cologne, Germany) with a standard preprogrammed protocol (A-PRF+ mode, 8 min, 1300 rpm). Patient blood was collected immediately before skin graft transplantation. Depending on the size of the defect, four to eight sterile glass-based vacuum tubes (10 mL) were used. After centrifugation, the blood clots were formed into membranes (solid PRF). The application of PRF membranes to the wound bed is illustrated in Figure 2.

### 2.3. Photographic Coverage Rate Analysis

**Figure 3:**
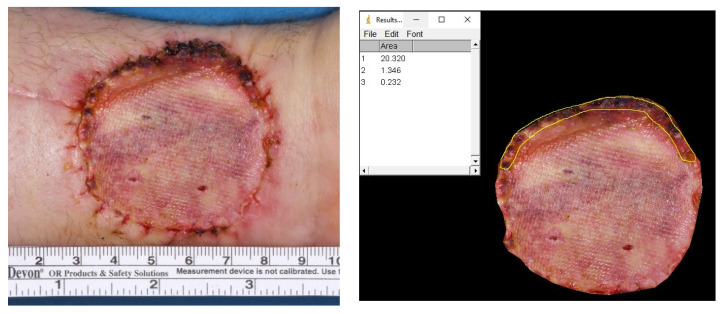
The left image depicts the photographic documentation after removal of the pressure bandage ten days after skin graft transplantation. On the (**right**) image, the surface area and area of dehiscence and necrosis were determined with the ImageJ software and with the help of the scale bar see (**left**) image. In the presented example, the area of the donor site was determined as being 20.320 cm^2^ and the area of graft loss as 1.578 cm^2^ (1.346 cm^2^ + 0.232 cm^2^). This led to a calculated coverage rate of 92.23% and a corresponding dehiscence rate of 7.77%.

To compare healing success and quantify transplant survival, photographs of the forearm were taken at time point T1 (suture removal). These photographs were imported into the software ImageJ (version 1.53a, Research Services Branch of the National Institute of Mental Health, National Institutes of Health, Bethesda, MD, USA) and analysed. We measured the surface area of the lesion in square centimetres and determined the coverage rate in square centimetres and percent (see Figure 3). An experienced maxillofacial surgeon (one of the authors) performed the evaluation in a single-blinded manner.

### 2.4. Evaluation Score

The same investigator who performed the photographic analysis determined the evaluation score for every patient in both groups (PRF vs. non-PRF) after ten to twelve days (time point T1, suture removal) in a single-blinded manner. The score was composed of complications such as infection, tendon exposure, or graft loss (please refer to Table 1 for the full score). Each item scored one point for “yes” and zero points for “no”, with the total loss of the graft scoring the maximum of six points. A higher score thus indicated worse graft in-growth and greater wound-healing impairment.

### 2.5. Statistical Evaluation

Statistical evaluation was performed using GraphPad Prism (version 8.53, GraphPad Software, San Diego, CA, USA) and an unpaired one-tailed and two-tailed *t*-test.

## 3. Results

### 3.1. Descriptive Statistics

Thirty-two patients with donor-site morbidity on the arm after harvesting a radial forearm flap were included in this study. The mean age of the patients was 62.03 (SD ± 18.98), with 21 males versus 11 females included in the study (Table 2 portrays detailed characteristics of all the participants).

Reconstruction of the donor site was performed either with a split-thickness skin graft from the thigh (*n* = 14) or with a full-thickness skin graft from the abdomen or arm (*n* = 18). Closure of the donor site was performed primarily after a radial forearm flap surgery in 13 patients, and in a second operation within two weeks after the main surgery for the remaining 19 patients (for example, tumour resection and reconstruction with microvascular radial forearm flap). Most patients received intravenous antibiotics during the course of the tumour resection without any effect on the healing or infection rates (see Table 3). Seven patients with secondary reconstruction did not receive any antibiotic therapy, because the perioperative prophylaxes after the tumour resection had already been finished.

### 3.2. Coverage Rate and Extent of Dehiscence

**Figure 4:**
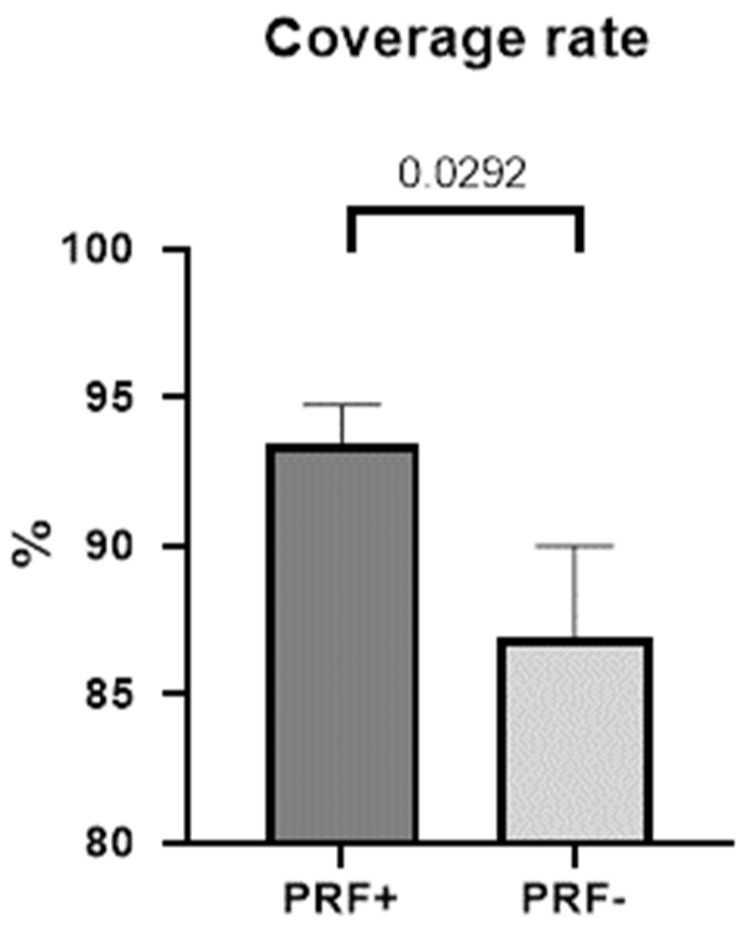
Coverage rate in percent in the PRF and non-PRF groups.

Photographic analysis with ImageJ was performed as described above (Section 2). This measurement revealed a mean coverage rate of 93.44% (SD ± 5.212) in the PRF group. In contrast, in the non-PRF group, the mean coverage rate was 86.96% (SD ± 12.10). The difference between both groups was 6.112% (SD ± 3.384), which was found to be statistically significant (*p* = 0.0292, 95% CI −0.2450–13.21) in an unpaired and one-tailed *t*-test (Table 4 and Figure 4).

The graph illustrates a mean coverage of 93.44% (SEM ± 1.303) in the PRF group and 86.94% (SEM ± 3.026) in the non-PRF group. The difference between both groups was statistically significant with an unpaired, one-tailed *t*-test (*p* = 0.0292).

### 3.3. Evaluation Score

**Figure 5:**
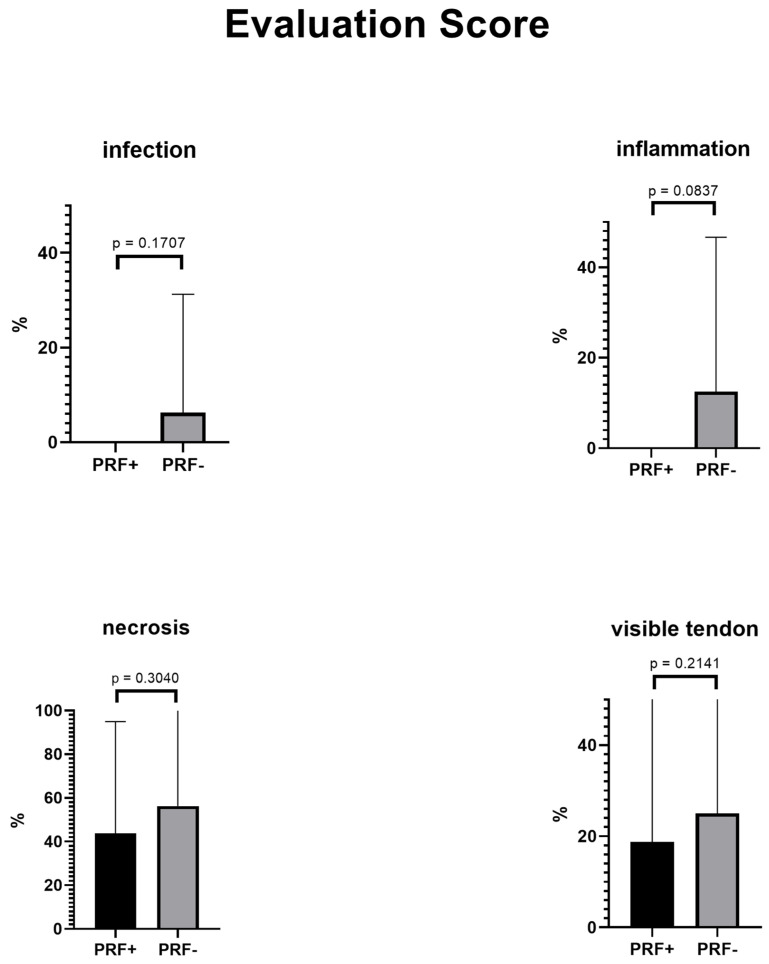

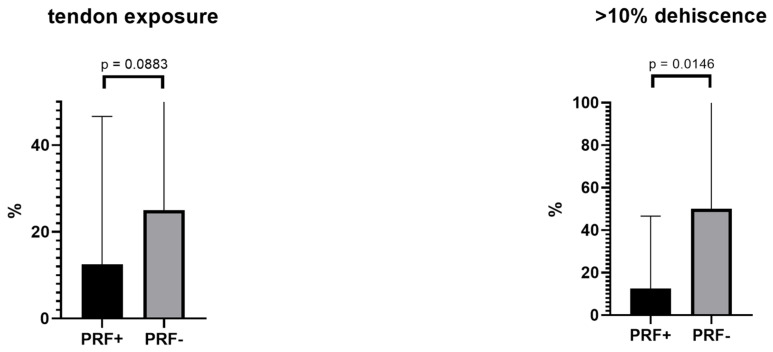
Evaluation score in the PRF group and in the non-PRF group.

The mean evaluation score was 1.5 in the PRF group and 2.688 in the non-PRF group. An unpaired, one-tailed *t*-test revealed a statistically significant difference between the evaluation scores (Table 5 and Figure 5) of both groups (*p* = 0.0458).

Single items in the score (infection, inflammation, necrosis, visible tendon, and tendon exposure) failed statistical significance but returned generally better results in the PRF group. Overall, no infection or inflammation was detected in the PRF group (Figure 5). The item >10% dehiscence was statistically significant (*p* = 0.0108). Thus, less major graft loss was detected in the PRF group. In both groups, no patient scored the maximum six points (total loss of the skin graft).

The mean difference between both groups was 1.188 (SD ± 0.6814, 95% CI interval: −0.2041–2.579), which was statistically significant with a one-tailed and unpaired *t*-test. Generally, results were better in the PRF group; however, the majority of single items failed statistical significance. A statistically significant improvement was only seen in the PRF group for >10% dehiscence (see Table 5 and Figure 5).

## 4. Discussion

The closure of the donor site following radial forearm flap harvesting is a well-known procedure and is normally performed using split-thickness skin grafts [17]. However, this procedure is plagued by wound healing impairments such as partial graft loss, tendon exposure, or dehiscence [3,17]. In a study by Karimi et al., 16% of the patients participating suffered from partial necrosis, and 4% suffered from exposure to the palmaris longus tendon [2]. Richardson reported similar results with partial skin loss in 16% of patients and tendon exposure in 13% of patients. Wound closure was achieved with split-thickness skin grafts [4]. In addition, the use of full-thickness skin grafts did not improve these results. In a comparison of full-thickness and split-thickness skin grafts, Davis et al. detected partial skin graft loss (>5%) in 17% of patients, minor necrosis in 34.1% of patients, and tendon exposure in 21.28% of patients, which were even higher than the values reported by Karimi and Richardson. There was no significant difference between the two groups relating to graft thickness [5]. This is consistent with the results of the non-PRF group in our study and the reason why several studies investigated methods and closure techniques such as dermal substitutes, negative-pressure wound therapy, or full-thickness skin grafts to reduce these surgical impairments [3,17]. Therefore, the primary coverage of the wound bed with a bioresorbable dermal substitute (Hyalomatrix) followed by a split-thickness skin graft after 3 weeks achieved better results than the immediate reconstruction with full-thickness skin grafts [18]. In our study, we were also able to show positive results for additional treatment of the wound bed with the insertion of PRF membranes.

Many of these methods should improve wound-bed conditions by enhancing granulation and capillary sprouting as a precondition of graft survival. However, most of these preconditioning methods need devices and treatments lasting several days, resulting in higher costs and increased patient stress [19,20]. Our study investigates a protocol that is simple, inexpensive, convenient, and effective at reducing complications as described above [3].

We already mentioned that the application of PRF is often indicated at damaged sites to reduce wound-healing complications. Several studies have already demonstrated that PRF can improve surgical outcomes, especially when wound healing is affected by poor blood supply (as in patients with osteonecrosis or compromised soft tissue) [16,21]. In the reconstruction of forearm defects, PRF membranes may serve as a source of growth factors and as an additional “lubricating” layer between tendons and the graft [9,10,22]. Compared to negative-pressure wound therapy, the use of PRF is simple, cost-effective, and does not require the long-term use of additional devices. Aside from the centrifuge, the costs for PRF preparation amount to a few euros. The results of this study reveal an indication of potentially better surgical outcomes in the PRF group compared to the non-PRF group. Based on the coverage rates and the evaluation score combining a number of associated surgical complications, the use of PRF indicates improved surgical outcomes. This is in line with several studies investigating the effects of PRF and platelet-rich plasma (PRP) in free skin graft survival [23,24]. Nica et al. investigated the effects of PRF on full-thickness skin grafts in a rat model. Analogous to our protocol, 40 rats were divided into two groups. In one group, PRF was applied to the wound bed before skin graft transplantation, and in the control group, the skin graft was directly placed on the wound bed. The results of this study showed less necrosis in the PRF group (14.9% ± 5.1) compared to the non-PRF group (28.5% ± 9.2) [25]. These results are consistent with those of our study and were supported by data from two meta-analyses, which concluded a positive effect of PRP in free skin graft survival [23,24]. PRP is a platelet-related product similar to PRF but without the firm fibrin structure and with the addition of anticoagulants [26].

The benefit of PRF in the survival of free skin grafts is possibly based on two major facts. Firstly, PRF is a source of growth factors, interleukins, and immune cells, which stimulate angiogenesis and the in-growth of vessels in the donor site and surrounding area [9]. Therefore, the stimulating effect of PRF on vessels and angiogenesis may improve the sprouting of new capillaries to perfuse the skin graft. As free skin grafts depend on a sufficient blood supply from the surrounding tissue, this is a crucial point in graft healing [6]. Furthermore, angiogenesis and PRF itself enhance the migration of inflammatory cells, as well as fibroblast proliferation and differentiation, which likewise supports wound healing and graft integration [27,28,29,30].

Secondly, the application of PRF between tendons and the skin graft may reduce mechanical stress on the graft triggered by movements of the hand (Figure 2). Particularly, flexion and extension of the hand cause the movement of the palmaris or flexor carpi radialis tendons, which can impair graft healing. A layer between these structures, acting as a lubricant, could reduce graft thinning and tendon exposure as well as tendon adherence to the graft, especially in cases of loss of the tendon fascia. Attempts to reduce this mechanical stress by using full-thickness skin grafts instead of split-thickness skin grafts have failed so far. Actually, the thicker transplants are more resistant to the tendon motion, but insufficient blood supply may negate these benefits [5]. The use of PRF may allow the combination of both (1) increased mechanical resistance and (2) sufficient blood supply by enhancing angiogenesis and fibroblast migration.

The application of PRF is simple and cost-effective. Wound complications, in particular, require further treatments such as local wound care or additional procedures, which may prolong hospital stays and reduce patient quality of life. This also supports the use of PRF and underlines the economic benefit.

Generally, wound infection does not seem to be the main complication in free skin graft transplantation. Indeed, there was only one infection detected in both groups. This infection occurred in the non-PRF group under intravenous antibiosis (Unacid). This is in line with the complications reported in the literature [2,4,5].

Limitations of the study include the small sample size and the implementation of two skin graft techniques (full-thickness vs. split-thickness) instead of only one, as well as the two time points at which reconstruction occurred (primarily vs. secondarily). In the literature, full-thickness and split-thickness skin grafts are described as equally appropriate for the reconstruction of the donor site following radial forearm flap surgery [5]. However, both aspects (time of reconstruction and thickness of the free skin graft) were not balanced in the PRF and non-PRF groups, which weakens the significance of our results. However, we do not expect this to be a major limitation of our findings. On the contrary, we believe that this reflects daily clinical routine (choice of harvest area is dependent on the preference of the surgeon and patient). Furthermore, it underlines the finding that the use of PRF is beneficial in different techniques of closure following radial forearm flap harvesting. To increase the evidence of the results and in order to exclude possible misjudgments or a selection bias, the evaluation could have been performed by two investigators in consent.

## 5. Conclusions

The application of PRF to the wound bed prior to free skin graft transplantation can improve surgical outcomes and coverage rate. With the supportive effect of PRF, we observed an indication of potentially better wound healing. For example, we observed a trend of fewer complications such as tendon exposure, dehiscence, and necrosis in the PRF group compared to the non-PRF group. The results of this study give a visible indication of the beneficial uses for the application of PRF in free skin graft transplantations to reconstruct the donor site following radial forearm flap harvest. Further clinical studies with larger case numbers and multicentre approaches are necessary to obtain a clearer indication.

## Figures and Tables

**Table 1:** Evaluation score.

		Yes	No
Total score	0–6		
Infection		1	0
Inflammation		1	0
Necrosis		1	0
Visible tendon *		1	0
Tendon exposure *		1	0
Transplant loss > 10%		1	0
Total transplant loss **		6	0

* “Visible tendon” was defined as tendons of the hand being seen through the skin graft as a predictor of graft loss in this area. “Tendon exposure”, in contrast, indicated dehiscence of the graft above the tendons. ** “Total transplant” loss was defined as critical point. The occurrence of this event scored the maximum number of points (6).

**Table 2:** Descriptive statistics.

	PRF+	PRF−
Age (±SD)	63.50 ± 18.04	62.38 ± 20.49
Participants (*n*)	16	16
Gender		
Women	5	6
Men	11	10
Diagnosis		
Oral cancer	12	16
Skin cancer	4	0
Graft size (cm^2^; ±SD)	19.5 ± 9.14	18.4 ± 5.95
Skin graft (*n*)		
Full-thickness	6	12
Split-thickness	10	4
Reconstruction		
Primary	4	13
Secondary	12	3
Intravenous antibiotics		
No antibiotic therapy	5	2
Ampicillin/sulbactam	11	13
Clindamycin	0	1

**Table 3:** Effect of intravenous antibiotics on the healing rate.

	Patients	Dehiscence in %	Infections	*p* (Dehiscence)
Intravenous antibiosis				0.3888 *
None	7	7.16	0	
Ampicillin/sulbactam	25	10.77	1	
or clindamycin				

Twenty-five patients underwent intravenous antibiosis (ampicillin/sulbactam or clindamycin in case of allergies), and the remaining seven patients did not undergo antibiosis. * There was no difference between the two groups regarding the occurrence of dehiscence and infection.

**Table 4:** Dehiscence: PRF vs. non-PRF.

	M (±SD) in %	Md	Range	*p* *	95% CI
Coverage rate					
PRF+	93.44 ± 5.212	94.73	80.92–100		
PRF−	86.96 ± 12.10	89.26	54.81–100		
Difference	6.483 ± 3294			0.0292	−0.2450–13.21

* Statistical significance for *p* < 0.05.

**Table 5:** Detailed comparison of the evaluation scores in the PRF and non-PRF groups.

	PRF	Non-PRF	Difference	*p*-Value
Mean total score	1.5	2.688	1.188	0.0458
Infection	0	1	1	0.1627
Inflammation	0	2	2	0.0768
Necrosis	7	9	2	0.2477
Visible tendon	3	4	1	0.3405
Tendon exposure	2	4	2	0.1907
>10% dehiscence	2	8	6	0.0108

Table 5 depicts the number of “yes” answers in each category. A higher evaluation score indicated a negative surgical result. The results were generally better in PRF group than in non-PRF group, but failed to be statistically significant apart from the item “>10% dehiscence”.

## Data Availability

The raw data are pseudonymised and available from the corresponding author on reasonable request.
